# 亚洲人面部注射年轻化和美学形象整体设计新方案

**DOI:** 10.1093/asjof/ojab011

**Published:** 2021-03-13

**Authors:** 崔 海燕, 赵 海光, 徐 海淞, 王 国宝, 和 谭琳琳

## Abstract

越来越多的亚洲人正在寻求非手术面部美容治疗。亚洲人的容貌、审美和解剖特征与西方人并不完全相同，同时还缺少系统化的美学评估和解决方案。作者从中国书法中吸取灵感，提出“未来”(Future Codes) 设计理念，来描述亚洲人面部美容注射艺术，从而帮助医生做好面部美容的设计与治疗。“未来”是由两个汉字组成的象形文字，翻译成英文就是“Future”，它代表着美好未来的意义，生动地描述了审美评估、设计及操作的方法。这一概念涵盖了亚洲人面部注射艺术的系统性总体设计，而且这些程序容易学习，执行起来也很安全。这是临床上第一个系统性解决方案，是通过东方哲学和文化针对亚洲人面部美化年轻化而提出的创新理念。

**证据等级：5:**

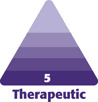

在全世界范围内，过去十年里，面部美容的非手术治疗量日益增多。许多患者选择皮肤填充剂和肉毒毒素来改善自己的容貌和修复组织缺失。^1^

在亚洲，面部美容的非手术治疗量也在增加。^7^虽然亚洲人群在文化、解剖结构、衰老程度和审美方面和 西方有着明显的不同，但是目前大多数面部注射方案的研究都是

肉毒毒素和皮肤填充剂是全球最流行的治疗年龄相关变

化的两种非手术性美容治疗，通常结合使用。^2^ 用于容量填充的填充剂有多种，如透明质酸 (HA)^3^、胶原蛋白、聚左旋乳酸^4^和羟基磷灰石钙。^5,6^ 这些填充剂可用于脸颊和下 巴的填充、鼻整形、脸中部丰盈和丰唇。为了减少皱纹的出现，很多人选择注射肉毒毒素。肉毒毒素可放松面部的某些肌肉，使皱纹在一段时间内不那么明显。^7^

针对西方人群的。^8,9^ 所以，亚洲人和白种人在治疗结果 和期望值方面均存在许多差异。为了指导正确地进行面部美容治疗，需要对亚洲面孔的主要美学关注点和要求有一个透彻的了解。现有的针对西方人群的指南不应该直接用于亚洲人。因此，需要有适合亚洲人群的治疗方案和设计理念。

大多数亚洲患者，无论年龄大小，都倾向于尽量避免手术，并寻求更加自然的效果。因此，应评估患者的期望，并提出针对亚洲人面部美化年轻化的创新理念，以满足亚洲患者的审美需求，包括脸型、结构、比例，以及衰老对其面部的影响。东方人和西方人的审美标准是不同的。西方人面部轮廓清晰，颧骨狭窄，轮廓结构分明，光影效果明显。东方人的脸型丰满，颧骨大，轮廓不清，皮肤细腻（图 1）。在西方国家，面部填充通常用于恢复活力和抵抗衰老，而东方人专注于填充和轮廓整形。由于亚洲人在面部形态和解剖结构上与西方人不同，因此无创性美容治疗是合适的。^7^然而，到目前为止，关于面部注射的大多数研究和建议都针对西方人群。 ^10-12^但是，无论民族和文化差异如何，某些美学原则，包括对称、平衡、比例、协调和多样性统一，都是东西方人群的共同点（图 1）。除了这些基本原则外，以下特征通常也被认为是讨人喜欢的：(1) 优雅的体态，(2) 苗条的身材，(3) 美丽的脸庞，(4) 细长的脖子，(5) 匀称的乳房，(6) 纤细的腰肢，(7) 圆润的臀部。^7,13^ 作者提出医学美容的三重境界：1. 掌握基本知识、操作技巧和解剖，熟练使用各种产品、设备和技术 — 解决基本问题；2. 轻松驾驭面部不同部位的操作，有效预防和处理并发症 — 解决复杂问题；3. 最高水平是把医学美容看成是医学限制条件下的艺术创作 — 发掘创造求美者潜在的、个性化的生动之美。

**图 1. (A):**
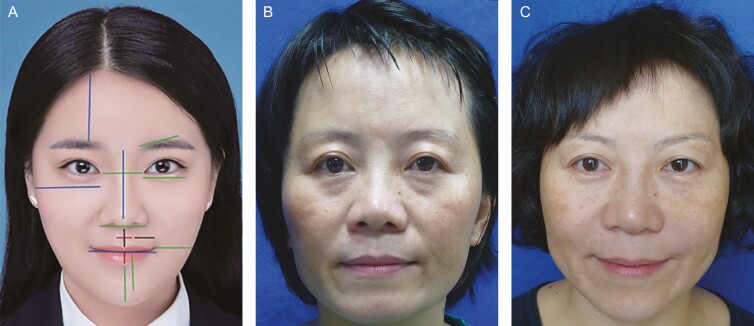
美丽的亚洲人面孔（26 岁女性）上的比例标记。绿线为基准 X，蓝线为 1.618 X，黑线为 0.618 X，橙线为 0.382 X，所有颜色相同的线条都具有相同的长度；1.618、0.618 和 0.382 均为黄金比例（阿瑟•斯威夫特博士的比例标记理论）。^14^ (B, C) 女性，48岁，注射前（左下）和注射后 3 个月（右下） — 采用“崔氏”(Cui Codes) 设计方法。

研究人员发现，人类对外表美的感知与黄金比例（约 1.618）密切相关。面部某些区域的长度与另一个特定区域的长度的理想比值为 1.618。^15^

大多数被认为是美丽或迷人的面孔都有大量的标记，其比例非常接近黄金比例。如图 1B 所示，绿线为基准 X，蓝线为 1.618 X，黑线为 0.618 X，橙线为 0.382 X；所有颜色相同的线条都具有相同的长度；各条线之间存在黄金比例。因此，比例美学是美的一个重要特征，在形式美学中占有重要地位。^14^

作者^16^于 2007 年发表论文，提出人体美学形象整体设计的理念，其中包括在治疗过程之前进行系统的总体设计和美学评估、在围疗期进行心理咨询，以及多种方法的综合运用。经过治疗后，化妆、服装、造型和礼仪训练是必要的补充，以创造散发魅力和活力的美丽容颜。

作者提出医学美容的三重境界：1. 掌握基本知识、操作技巧和解剖，熟练使用各种产品、设备和技术 — 解决基本问题；2. 轻松驾驭面部不同部位的操作，有效预防和处理并发症 — 解决复杂问题；3. 最高水平是把医学美容看成是医学限制条件下的艺术创作 — 发掘创造求美者潜在的、个性化的生动之美。

在提倡生物-心理-社会医学模式的同时， 除了外科技术外，还需要医生的审美感知和人文关怀，尤其是在整形外科领域。美容注射应遵循整体设计的原则。对于一些患者来说，即使手术完成得很好，结果却并没有令患者满意，因为他们的医生只解决了局部问题，而忽视了局部和整体的关系。^7,13^ 例如，一个完美的鼻子必须与整张脸有很好的比例美学关系，同时还要考虑到矫正鼻唇沟的患者初衷。注射后，鼻唇沟的问题解决了，但脸却变胖了。改善对审美的认识和人文关怀更加重要，对于整形外科医生尤为如此。^17^ 在进行美容注射之前，必须考虑人体美学形象的整体设计和构建，这样才能获得更好的治疗效果、心理满足感，以及患者的社会认可度。此外，按照生物-心理-社会医学模式，在整个治疗过程中应注意保持患者的心理状态稳定。

在总结数千个教学和培训案例的基础上，作者从中国书法中汲取灵感，提出“未来”（Future Codes）设计理念，来描述亚洲人面部美容注射的艺术，从而帮助医生做好面部美容的设计与治疗。“未来”（图 2A）是由两个汉字组成的象形文字，翻译成英文就是“Future”，它代表着美好的意义，生动描述了审美评估、设计及操作的方法。

**图 2.**
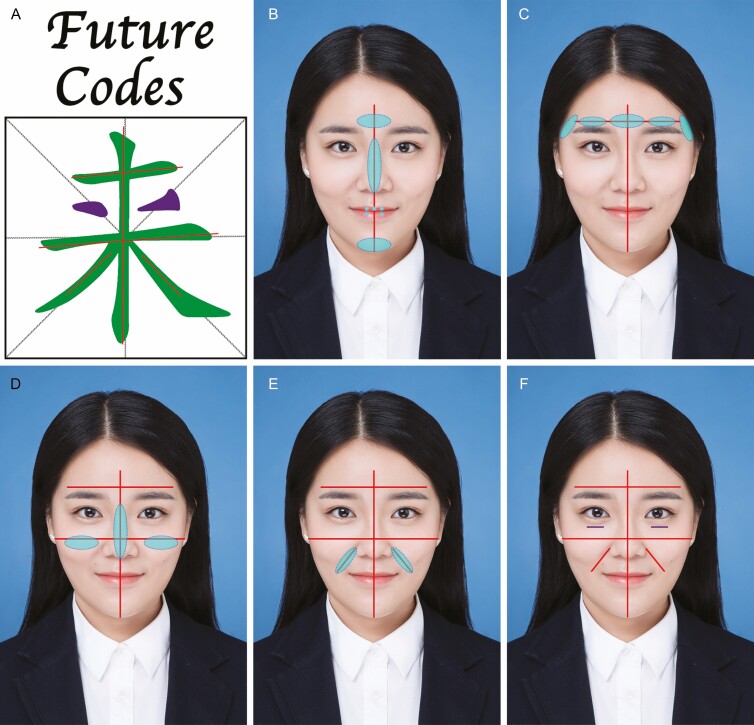
崔医生的“崔氏”设计基于中国书法。(A) 2 个汉字“未来”在英文中是“Future”的意思。这一概念涵盖了亚洲人面部注射艺术的系统性总体设计。(B) 面部中线（B，26 岁女性）穿过前额、眉间复合体、鼻子、嘴唇和下巴。(C) 第一条横线穿过眉弓、眉毛和眉间复合体。(D) 第二条横线穿过脸颊或“苹果肌”。(E) 另外两条斜线沿着鼻唇沟延伸。(F) 最后，加上 2 个鼻唇沟、泪沟。

“未来”描述了一个易于学习的系统性总体设计概念，可以帮助医生获得满意的结果。本文中所述的注射方法隐藏在两个汉字的中国书法中，由以下线条组成，反映了亚洲人面部的衰老特征（视频）。

面部中线（图 2B）穿过前额、眉间复合体、鼻子、嘴唇和下巴。这条线非常重要，因为它决定了脸部两侧的轮廓、对称性、平衡性、协调性、比例、立体感、光线和阴影，因此会影响整个手术过程的成败。因而这是“未来”设计理念中的重点。

第一条横线（图 2C）穿过眉弓、眉毛和眉间复合体。第二条横线（图 2D）穿过脸颊或“苹果肌”。如果在这条线内注射，颧骨会显得更窄，有助于减小亚洲人颧骨的宽度。眉毛和苹果肌的横线也被认为是衰老的标准。明显的泪沟和鼻唇沟被认为是衰老的迹象。另一方面，另外两条斜线沿着鼻唇沟延伸（图 2E）。 如果用注射填充剂处理鼻唇沟，患者会显得年轻。最后，添加了 2 个唇沟和泪沟（图 2F），将这两个汉字和“未来”结合在一起。“未来”设计理念包括面部轮廓的重要美学点和线，并包含面部年轻化和美学的核心原则（图 2）。所有这些线条构成了汉字“未来”。因此，这两个字的中国书法代表了亚洲患者美容面部注射的核心。

衰老的出现主要是由于皮肤和软组织松弛、组织结构移位、皮肤出现皱褶、皮肤纹理发生变化、组织容量和弹性不足所致。“未来”中也体现了这些特点。在解剖学知识的指导下，手术和注射是可行的，并且采用这种注射方法可以有效地避免并发症的发生。

## 案例展示

按照作者的 “未来”设计方法，一名 48 岁女性经历了以下注射过程：

1. 中线注入：(1) 将 1 毫升透明质酸注入眉间复合体，方法是用尖头针穿刺，直到针尖接触到骨膜，确保没有血液被抽出，然后缓慢推进。注射时按摩该部位。(2) 通过用 23G 钝头针穿刺鼻尖，向鼻子注射 1 毫升透明质酸，加 1% 利多卡因进行局部麻醉。(3) 用 30G 的尖锐针头向嘴唇注射总共 0.4 毫升的透明质酸：唇 3 处，下唇 2 处各注射 0.08 毫升。(4) 用一根 27-30G 的尖锐针头向下巴注射 1 毫升透明质酸，直到针尖到达骨膜。
2. 第一条横线的注入：(1) 用钝头针在眉尾皮下穿刺，用 23G 钝头针向注射 0.6 毫升透明质酸。透明质酸注射到，同时皮下针头。(2) 用尖头针将针尖接触到骨膜，向颞区注射 2 毫升透明质酸，确保没有血液被抽出，然后缓慢推进。在颞区两侧各注射 1 毫升透明质酸，同时进行按摩。
3. 向“苹果肌”的第二条横线注入 1 毫升透明质酸。对苹果肌、鼻唇沟、泪沟，用 23G 钝头针在距口角外 1.5 厘米处、皮下或骨膜处穿刺，每侧注射 0.8 毫升透明质酸。针尖可以到达苹果肌、泪沟和鼻唇沟。
4. 从口角外 1.5 厘米处穿刺，向鼻唇沟注射 2 毫升透明质酸，两侧各注射 1 毫升。
5. 从口角外 1.5 厘米处穿刺，向泪沟注射 0.3 毫升透明质酸，两侧各注射 0.15 毫升。

### 辅助治疗

注射治疗后，还可以采用以下步骤，以达到更好的效果：(1) 可用光纤激光溶解眼袋脂肪；(2) 可用射频收紧嘴角和下颌边缘的皮肤；以及 (3) 可以修剪头发。

### 结果

通过系统全面的“未来”设计，患者获得了一张比例协调、轮廓分明、明暗对比清晰、皮肤紧致和眼袋扁平的脸。这些手术使人的眼睛看起来更加神采奕奕，鼻子结构立体自然，苹果肌圆润，颧骨狭窄，鼻唇沟浅圆，下巴的长度、凸度和半径得以增大。整个面容看上去更年轻（图 1D，补充图 1 和 2）。

## 声明

上海同济大学同济医院制度审查委员会医学伦理委员会对此研究进行了伦理批准。并且根据法规，为每个参与者获得了书面知情同意书。

## 结论

越来越多的亚洲人正在追求面部美容的非手术治疗。亚洲人的容貌、审美和解剖特征与西方人并不完全相同，同时缺少系统化的美学评估和解决方案。作者提出医学美容的 

三重境界：1. 掌握基本知识、操作技巧和解剖，熟练使用各种产品、设备和技术 — 解决基本问题；2. 轻松驾驭面部不同部位的操作，有效预防和处理并发症 — 解决复杂问题；3. 最高水平是把医学美容看成是医学限制条件下的艺术创作 — 发掘创造求美者潜在的、个性化的生动之美。基于这些理念，作者从中国书法中汲取灵感，提出“未来”(Future Codes) 设计理念，来描述亚洲人面部美容注射的艺术，从而帮助医生做好面部美容的设计与治疗。这一概念涵盖了东方审美、东方解剖特征、东方评估审美解决方案，是亚洲人面部注射艺术的系统性总体设计。作者

崔海燕医生有 10,000 多例治疗经验，并且具有丰富的教学和培训经验。该方案操作简便，易于临床医生掌握。这是临床上第一个系统性解决方案，是通过东方哲学和文化针对亚洲人面部美化年轻化而提出的创新理念。

## Supplementary Material

ojab011_suppl_Supplementary_Figures

## 披露

作者声明在本文的研究、撰写和发表方面不存在潜在的利益冲突。

## 资助

作者在本文的研究、撰写和发表过程中没有获得任何资助。
